# Layer-by-Layer Pyramid Formation from Low-Energy Ar^+^ Bombardment and Annealing of Ge (110)

**DOI:** 10.3390/nano11102521

**Published:** 2021-09-27

**Authors:** Marshall van Zijll, Samantha S. Spangler, Andrew R. Kim, Hazel R. Betz, Shirley Chiang

**Affiliations:** 1 Department of Physics and Astronomy, University of California Davis, Davis, CA 95616-5270, USA; mvanzijll@eccc.edu (M.v.Z.); sspangler@micron.com (S.S.S.); arykim@ucdavis.edu (A.R.K.); hazel.betz@gmail.com (H.R.B.); 2 Department of Science, East Central Community College, Decatur, MS 39327, USA; 3 Micron Technology, Manassas, VA 20110, USA; 4 Intel Corporation, Hillsboro, OR 97124, USA

**Keywords:** Germanium (110), argon-ion sputtering, scanning tunneling microscopy, nanostructure formation, metal co-deposition

## Abstract

Isolated pyramids, 30–80 nm wide and 3–20 nm tall, form during sputter-annealing cycles on the Ge (110) surface. Pyramids have four walls with {19 13 1} faceting and a steep mound at the apex. We used scanning tunneling microscopy (STM) under ultrahigh vacuum conditions to periodically image the surface at ion energies between 100 eV and 500 eV and incremental total flux. Pyramids are seen using Ar^+^ between 200 eV and 400 eV, and require Ag to be present on the sample or sample holder. We suspect that the pyramids are initiated by Ag co-sputtered onto the surface. Growth of pyramids is due to the gathering of step edges with (16 × 2) reconstruction around the pyramid base during layer-by-layer removal of the substrate, and conversion to {19 13 1} faceting. The absence of pyramids using Ar^+^ energies above 400 eV is likely due to surface damage that is insufficiently annealed.

## 1. Introduction

Surface features formed through ion bombardment depend on many parameters, including sputter ion type, fluence, flux, energy, and incident angle; sample type, orientation, and temperature; as well as sample cleanliness and the presence and type of contaminants and defects. The methods of formation reported in the literature are varied, including dot and ripple patterns [1], faceted ripples [2], pyramids [3], positive-growth whiskers and cones [4], vacancy accumulation of pits [5], and many patterns that rely on an interplay of roughening and smoothing during high-energy ion bombardment [1,6,7,8].

This paper discusses the formation of isolated pyramids with {19 13 1} faceted sides that form during sputter-annealing cleaning cycles performed on the Ge (110) surface. Interesting optical and biological applications for pyramids such as these are discussed in other works [9,10,11].

Each cleaning cycle is 15 min of ion bombardment with the sample at 500–650 °C, followed by 10 min of annealing the sample to 800 °C. Usually, this process results in a Ge (110) surface with large, atomically flat domains of surface reconstructions—ideal for imaging with scanning tunneling microscopy (STM). Pyramids are only observed when performing cleaning cycles on two types of samples: Ge (110) dosed with 10 monolayers (MLs) of Ag, and bare Ge (110) using a sample holder that must still have had many MLs of Ag. In the absence of Ag, the surface becomes smooth from sputter-annealing, and no pyramid formation is observed (Appendix A). Thus, we infer that co-sputtering of Ag from the sample holder initiates the formation of the pyramids. We also found that pyramids may act as nucleation points for Ag one-dimensional (1D) island growth (see Appendix B).

Many effects are often associated with ion bombardment, such as collision cascade, sputtering, surface damage, ion implantation, amorphization of the surface, viscous flow, and ion reflection and re-deposition. Theories about pattern formation during ion bombardment at high temperatures typically discuss an interplay between roughening (due to bombardment and anisotropic surface diffusion) and smoothing (due to surface diffusion processes) [1,12,13,14,15]. In this experiment, the smoothing effect strongly outweighs the roughening effect. Comparing our cleaning cycle parameters to similar ion bombardment experiments [12,13,14,16] supports this. While other high-temperature experiments were typically carried out at 270–450 °C, our sample temperatures were even higher, 500–650 °C. For both high temperature ranges. the surface remains crystalline during bombardment. The annealing step, which is not performed in other experiments, further encourages a smooth surface. Other low-energy experiments typically use between 500 eV and 1 keV ions, but pyramids in this work only formed after sputtering using ions with energies between 200–400 eV. Other experiments typically used fluence ranging from 1 × 10^17^ to 1 × 10^20^ cm^−2^, showing that a sample becomes very rough at high fluences, but the surfaces in this experiment showed little change throughout the fluence range of 4.5 × 10^16^ to 1.8 × 10^18^ cm^−2^.

Simultaneous co-deposition of even very small amounts of metal during ion bombardment has been found to initiate different formations during ion bombardment, such as cones and whiskers [4,17,18,19,20] or nanodots [2,19,21]. While metal co-deposition during ion bombardment has primarily been studied at room temperature, in our experiments, Ag was co-deposited during high-temperature bombardment. Our experimental parameters caused our surface to become generally smoother following each cleaning cycle, and pyramids formed with well-organized walls that were flat at the atomic scale. The highly ordered crystal surface of the pyramids allows high-resolution STM measurements of their structure.

We used STM under ultrahigh vacuum (UHV) conditions to periodically image the surface at different ion energies and fluences. The evolution of the surface suggests that the pyramids are nucleated by Ag clusters co-sputtered onto the surface, and that the pyramids grow due to layer-by-layer removal of the substrate near the pyramid base.

## 2. Materials and Methods

All sample preparation and measurements were performed in an ultrahigh vacuum system consisting of three principal chambers housing a low-energy electron microscope (LEEM, Elmitec Elektronenmikroskopie GmbH, Clausthal-Zellerfeld, Germany), an STM (Oxford Instruments, Eynsham, UK), and an X-ray photoemission spectrometer (VG Microtech, East Grinstead, UK) [22]. Ge (110) samples were prepared from Sb-doped Ge (110) wafers (resistivity between 0.1 and 1.0 Ω-cm, 2 inches in diameter and 0.5 mm thick, with reported miscut <0.5°) purchased from MTI Corporation. Approximately 1 cm^2^ square samples were manually cut with a diamond scribe, rinsed in methanol and then hydrogen peroxide, before placing them into the STM–LEEM sample holder prior to insertion into the UHV chamber with a base pressure of $1\times 10^{-10}$ torr. The sample holders used were coated with many MLs of Ag from previous experiments. Some samples were coated with 10 MLs of Ag after placing them in the UHV chamber. In order to form pyramids, the samples were cleaned by sputter-annealing. Each cleaning cycle consisted of 15 min of sputtering followed by 10 min of annealing at 800 °C. After the sample cooled below 200 °C, we performed ion bombardment on the surface using Ar^+^ ions with energies ranging from 100 to 500 eV at an incident angle of 34° from the direction normal to the surface, with the sample continuing to cool during the ion bombardment. New samples were used for experiments with different bombardment energies. We imaged the sample every 5–7 cleaning cycles, and the samples went through a total of ~40 sputter-annealing cycles. The sputtering current could vary between 2 and 8 μA, depending on the energy, partial pressure of Ar, and the sample distance from the gun. Typical sputtering parameters for these measurements were Ar^+^ ion partial pressure of $5\times 10^{-5}$ torr, sputtering current of 2 μA, and an ion flux of ~1.25 × 10^13^ s^−1^ cm^−2^. The fluence ranged from 4.5 × 10^16^ to 1.8 × 10^18^ cm^−2^, but since we varied our ion energy between 100 and 500 eV, the fluence range varied for different ion energies. The direction of sputtering is incident from the right side when observing the STM images in this work. The top plate of the sample holder was machined from Mo (see Figures 3 and 5 from [18]). Following sputter-cleaning of either Ag/Ge (110) or bare Ge (110), XPS revealed no Ag on the Ge surface at detectable levels.

## 3. Results

**Figure 1:**
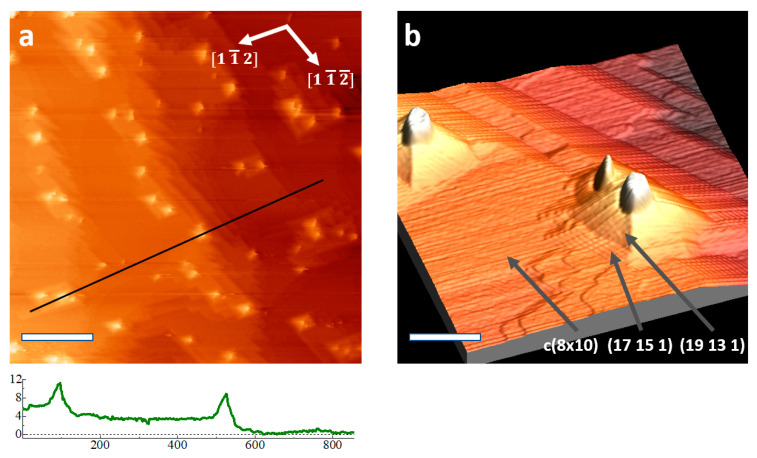
Scanning tunneling microscopy (STM) image of a Ge (110) sample that was coated with 10 MLs of Ag, followed by 10 cleaning cycles performed using 250 eV Ar^+^ ions. Imaging parameters 0.5 nA, 2 V tip bias. (**a**) Topographical image with profile (units in nm) taken along line shown below; (**b**) 3D image (magnified near center of (**a**)). Fluence: 4.8 × 10^17^ cm^−2^. Scale bars: (**a**) 200 nm, (**b**) 40 nm.

Examples of pyramid formations on the Ge (110) surface are shown in Figure 1. Pyramids have four walls parallel to the $\left[1\;\bar{1}\;\bar{2}\right]$ and $\left[1\;\bar{1}\;2\right]$ directions, at an inclination near 10.9° with respect to the flat substrate; these walls are parallel to the {19 13 1} faceting. A small mound with steep sidewalls is present at the apex of each pyramid; these are the bright features in Figure 1a. Surrounding the pyramids are flat terraces exhibiting the c (8 × 10) reconstruction of clean Ge (110), and dense steps typically formed from the (16 × 2) reconstruction [23]. Closely stepped (16 × 2) reconstruction forms {17 15 1} faceting, which is abundant on these surfaces [23]. The c (8 × 10) surface reconstructions and {17 15 1} faceting are also found on cleaned Ge substrates not showing pyramids, but the density of {17 15 1} faceting here is higher than is typical compared to Ge (110) cleaned without Ag present. The {19 13 1} faceting is a new observation, and a model is provided below.

Positive surface features such as these pyramids are expected to be stable under ion bombardment due to reduction of sputtering at local topographical maxima and enhanced sputtering at local minima [24,25,26,27]. The steep features observed near the apex of these pyramids were predicted by Sigmund to be a result of sputtering; the shape near the apex should become steep due to ion impact, since sputtered atoms are located “downstream” from the point of ion impact [24].

In contrast to the stability of pyramids, step edges change location during the sputter-annealing process. On a gradually stepped surface without these pyramids, our sputter-annealing parameters would typically cause step edges to recede in the direction of the higher terrace due to anisotropic surface diffusion combined with layer-by-layer removal of the substrate [5,7,18,28,29,30].

We suspect that the pyramids are immobile upon nucleation, and their locations inhibit the movement of step edges; this results in bunching-up of step edges, and the formation of local {17 15 1} faceting. While the four walls of the pyramids are typically similar in size, the {17 15 1} faceting generally forms long sections perpendicular to the sputtering direction (incident from the right side of all STM images shown). Additionally, the faceting forms along paths bordering pyramids. In many cases, the faceting wraps closely around pyramids and groups of pyramids, and examples of this are present on the right half and the bottom of Figure 1a. Despite the proximity of the pyramids to the faceting, no pyramids are formed with {17 15 1} walls.

**Figure 2:**
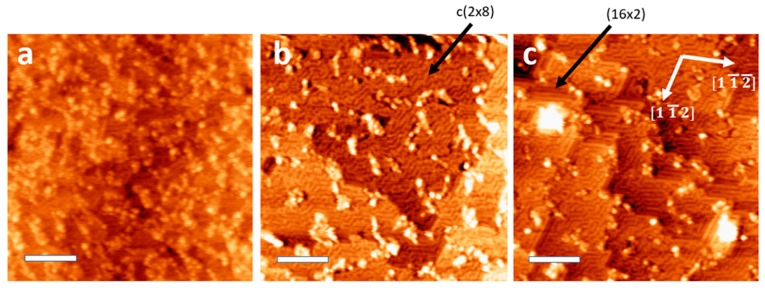
STM topographical images of a bare Ge (110) sample with (**a**) 14 cleaning cycles, fluence 1.6 × 10^17^ cm^−2^; (**b**) 23 cleaning cycles, fluence 2.6 × 10^17^ cm^−2^; and (**c**) 33 cleaning cycles, fluence 3.7 × 10^17^ cm^−2^, performed in a Ag-coated sample holder using 100 eV Ar^+^. Imaging parameters 0.5 nA, 2 V tip bias. Scale bars: 30 nm.

Figure 2 shows surfaces sputter-annealed with 100 eV Ar^+^ after 14, 23, and 33 cleaning cycles. After 14 cleaning cycles, the surface is still rough on an atomic scale, with each small dot in Figure 2a likely representing a small cluster of Ge adatoms. After 23 cleaning cycles (Figure 2b), the smoothing effects of sputter-cleaning become apparent, the surface reconstruction is predominantly c (8 × 10), and the step edges have intermittent straight sections. The (16 × 2) reconstruction is not obviously present on this surface. After 33 cleaning cycles, many step edges exhibit (16 × 2) reconstruction and align with the $\left[1\;\bar{1}\;\bar{2}\right]$ and $\left[1\;\bar{1}\;2\right]$ directions; this surface reconstruction is mostly found near step edges. The (16 × 2) surface reconstruction is most often observed forming long rows, and this characteristic may contribute to the formation of regular straight sections along the step edges [23,31].

Figure 2c also shows two large clusters on the surface, although from topographical images it is unclear whether these adatoms are Ge, Ag, or other contaminants. Since pyramids only form with Ag present, we suspect that these clusters contain Ag. The clusters are propped up on small terraces around the same size as the clusters and are located near step edges similar to pyramids at higher energies. These are basically pyramids that are only one or two atomic layers tall, with a cluster at the apex. It is possible that continued sputtering with this energy would eventually form large pyramids, but the rate of growth would be very slow. The appearance of these pyramid-like features coincides with straightened step edges forming the (16 × 2) reconstruction.

**Figure 3:**
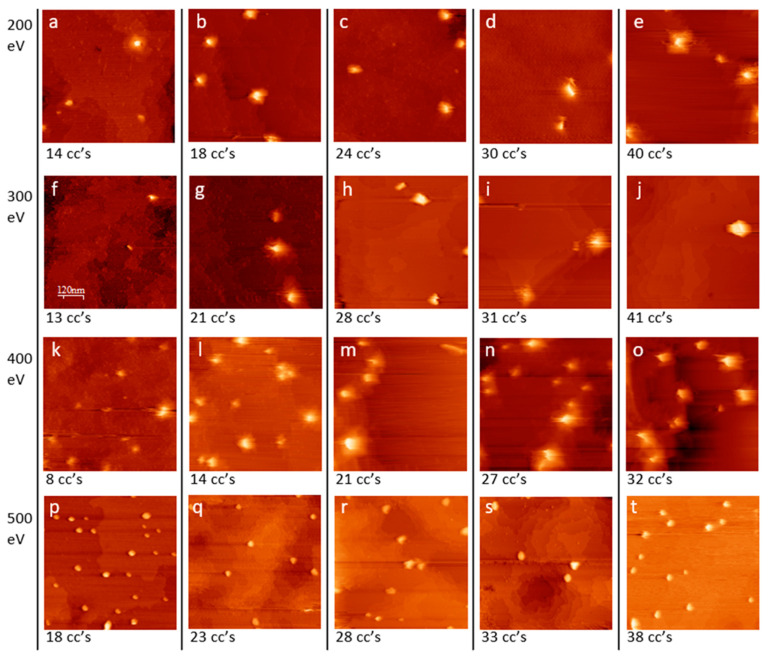
STM topographical images of bare Ge (110) samples with number of cleaning cycles (cc’s) shown beneath each image. Cleaning was performed in Ag^−^ coated sample holders using Ar^+^ with energies and fluence ranges of (**a**–**e**) 200 eV, 2.8 × 10^17^ cm^−2^ to 7.9 × 10^17^ cm^−2^; (**f**–**j**) 300 eV, 3.7 × 10^17^ cm^−2^ to 1.2 × 10^18^ cm^−2^; (**k**–**o**) 400 eV, 2.9 × 10^17^ cm^−2^ to 1.2 × 10^18^ cm^−2^; and (**p**–**t**) 500 eV, 8.1 × 10^17^ cm^−2^ to 1.7 × 10^18^ cm^−2^. Imaging parameters 0.5 nA, 2 V tip bias. Image sizes (**a**) 300 nm × 300 nm; (**b**–**t**) 600 nm × 600 nm.

When the sample is bombarded with 200 eV ions (Figure 3a–e), isolated pyramids are found on the surface. With 14 cleaning cycles (Figure 3a), only very small pyramids were found, but with 18 or more cleaning cycles, the surface bombarded with 200 eV ions appeared similar. Locations without pyramids were covered by atomically flat terraces with surface reconstructions of bare Ge. Using 300 eV ions (Figure 3f–j), resulted in images similar to those seen using 200 eV. Using 400 eV ions (Figure 3k–o), pyramids are more densely grouped on the surface, and some pyramids are larger than those seen using lower energies. Using 500 eV ions (Figure 3p–t), small mounds form on the surface that are different from the pyramids formed with lower energies.

In general, Figure 3 shows very little change to the surface with increasing fluence (2.8 × 10^17^ cm^−2^ to 1.7 × 10^18^ cm^−2^) for any given ion energy. The surface changes the most with lower numbers of cleaning cycles, because new samples are rough, as shown in Figure 2, and the first cleaning cycles flatten these surfaces.

Once the surface forms large surface domains, which typically takes 8–16 cleaning cycles, additional cleaning cycles cause fewer changes to the surface. This is in contrast with other ion bombardment experiments, where increasing fluence caused an increase in surface roughness [12,14,15,16,32].

**Figure 4:**
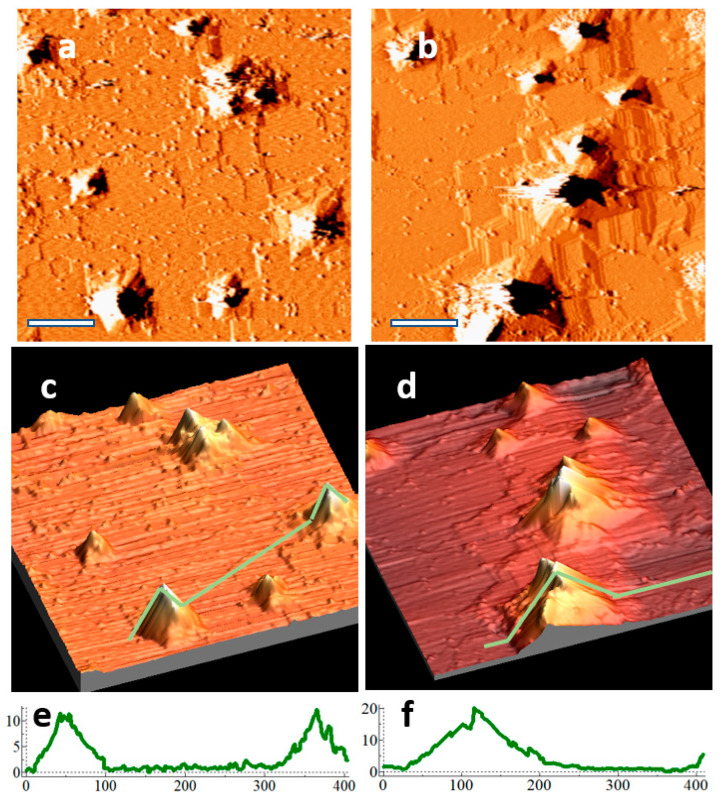
(**a**) STM derivative image of a bare Ge (110) sample in a Ag-coated sample holder after 14 cleaning cycles, fluence 5.1 × 10^17^ cm^−2^; (**b**) STM derivative image of the same sample after 27 cleaning cycles, fluence 9.9 × 10^17^ cm^−2^; (**c**,**d**) 3D views of (**a**,**b**), respectively, with associated line profiles along the green lines shown in (**e**,**f**), respectively.

Figure 4 shows representative areas on a sample that has been sputtered with 400 eV ions for 14 and 27 cleaning cycles. With fewer cleaning cycles, typically only small pyramids were found on the surface. At higher numbers of cleaning cycles, both large and small pyramids were found on the surface. The combination of large and small pyramids is present after 27 cleaning cycles in Figure 4b,d,f, suggesting that the initiation of pyramids is an ongoing process, presumably due to the constant supply of Ag seeding material from the sample holder. Since pyramid density remains nearly constant with increasing fluence, some pyramids are likely removed from the surface during the cleaning cycles to offset the formation of new pyramids.

The step density of the substrate was higher on the sample that underwent more cleaning cycles. The location of steps near the pyramids supports two hypotheses: (1) pyramids inhibit local terrace removal, and (2) a component of pyramid growth is through pinning of step edges at the pyramid’s base. Even though the step edges form {17 15 1} faceting when further from the pyramid, they must convert to {19 13 1} in order to contribute to pyramid growth.

Pyramids are often clustered together, suggesting that pyramid locations may encourage the initiation of others nearby. We suspect that pyramids inhibit the local rate of terrace removal and, thus, cause the bunching of steps; nearby locations at the top of bunched-up steps might be ideal for the initiation of new pyramids. Contaminant clusters that initiate pyramids at these locations may experience quicker growth, since sputtering of nearby step edges can quickly contribute to their height. This may enhance the stability of newly formed pyramids against disintegration.

Scanning artifacts are present in many of the STM images, where the scanning tip often “jumped” near the apex. This was common on pyramids formed when Ag was co-sputtered from the sample holder, but pyramids formed following sputter-cleaning of Ag-dosed samples were more often imaged clearly. This may be related to the steep slope, roughness, contamination, or disorganization of the peaks.

**Figure 5:**
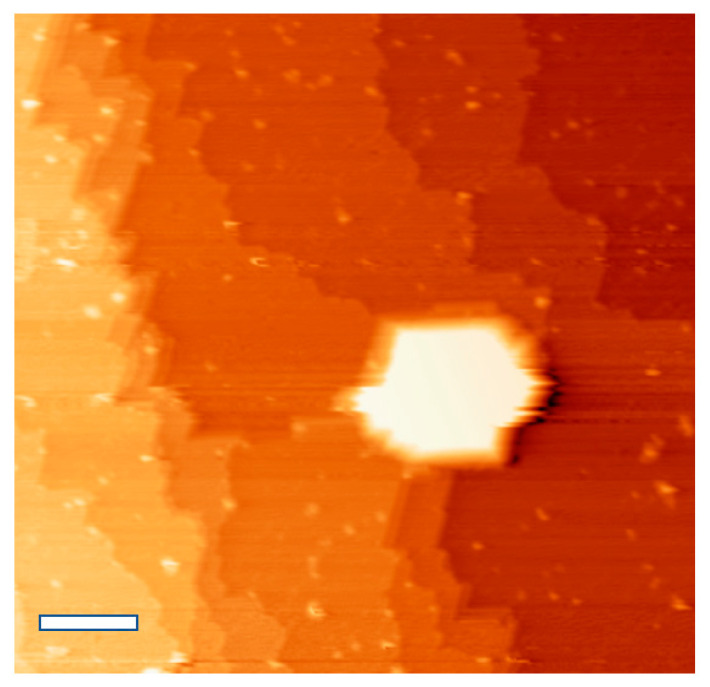
STM topographical image of a bare Ge (110) sample with 18 cleaning cycles, fluence 3.5 × 10^17^ cm^−2^, performed in a Ag-coated sample holder using 200 eV Ar^+^ ions. Imaging parameters 0.5 nA, 2 V tip bias. The contrast is enhanced to show the step edges, making the central pyramid appear to be saturated in brightness. Scale bar: 50 nm.

Figure 5 shows a pyramid located near the center of the image, with Ge (110) terraces at different heights. To the left of the pyramid, the step edges are densely spaced and are nearly perpendicular to the sputtering direction. At the location of the pyramid, however, step edges appear to be pinned at the pyramid’s perimeter. Higher step edges wrap increasingly more around the pyramid compared to lower step edges. We suspect that the general movement of the step edges in this image, during sputter-annealing cycles, is to the left. However, step edge movement appears to be locally inhibited near the pyramid. Defects on a surface have been proposed to inhibit migration or Ostwald ripening of adatoms and vacancies [24,33], and the pyramid here appears to be an example of such a defect. We propose that, as each step edge moves past the location of the pyramid due to subsequent sputter-annealing cycles, the pyramid grows in height by one atomic layer.

**Figure 6:**
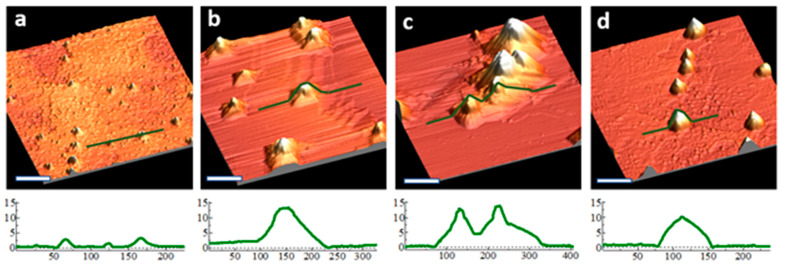
STM 3D images of a bare Ge (110) sample with cleaning cycles performed in a Ag-coated sample holder using (**a**) 100 eV Ar^+^, 33 cleaning cycles, fluence 3.7 × 10^17^ cm^−2^; (**b**) 200 eV Ar^+^, 40 cleaning cycles, fluence 7.9 × 10^17^ cm^−2^; (**c**) 400 eV Ar^+^, 32 cleaning cycles, fluence 1.2 × 10^18^ cm^−2^; and (**d**) 500 eV Ar^+^, 38 cleaning cycles fluence 1.7 × 10^18^ cm^−2^. Profiles below each image, with units in nm, are taken along the lines shown in the image. Imaging parameters 0.5 nA, 2 V tip bias. Scale bars: 120 nm.

Figure 6 compares surfaces sputter-annealed using 100 eV, 200 eV, 400 eV, and 500 eV Ar^+^ ions. This figure shows a progression similar to that seen in Figure 2, but these images have higher resolution and include line profiles. Even though the cleaning cycles are not identical in Figure 6, we have shown (Figure 2) that differences in fluence do not cause significant changes to samples. In contrast to fluence, different bombardment energies do result in obvious differences in the surface formations.

The surface features grow larger with higher energies between 100 eV and 400 eV. Sputter-cleaning cycles performed using Ar^+^ energies between 200 eV and 400 eV caused four-sided pyramids to form, similar to those already discussed. The pyramids formed with 400 eV Ar^+^ were often larger than pyramids formed with lower energy Ar^+^ and had larger mounds at the apex. Similarly, the substrate sputtered with 400 eV Ar^+^ had longer inclines of densely packed step edges. The slopes of the walls of larger pyramids were sometimes different along one direction compared with the rest of the pyramid (see Figure 6c). This is likely a shadowing effect enhanced by the large pyramid size, but this effect does not seem to contribute to pyramid formation. In all of the images, the pyramid slopes were calculated, and, except for a few defects and shadowing cases, the slopes were consistent with {19 13 1} faceting.

Using 500 eV Ar^+^ ions (Figure 6d), mounds formed on the surface, and pyramids were not found. The mounds do not grow as large as the pyramids, but their appearance is similar to the mounds seen at the apexes of pyramids. In general, the mounds formed at 500 eV are similar in size to the mounds found at the apex of 400 eV pyramids. Interestingly, there is no faceting on these surfaces, nor are there densely packed step edges. Moreover, the borders of the step edges are curving with few straightened sections. The substrate is rougher and has more defects compared to lower energy sputtering, and there was no clear surface reconstruction.

## 4. Discussion

The high level of geometry on many of these surfaces is certainly assisted by the annealing process, which repairs the kinetic damage caused by ion bombardment [34]. Annealing reduces the surface free energy, encouraging the formation of flat terraces with organized surface reconstructions [5,18,27].

The low-energy Ar^+^ ions used in our experiments likely only interact with a few layers near the surface [7]. We can roughly estimate 1–2 layers with 100–200 eV (at these energies the low-energy electron diffraction (LEED) pattern at the surface is not disrupted), and 4–8 layers with 300–400 eV [35,36]. Ion interaction with these layers causes the creation of defects due to kinetic impact and collision cascades [5,24,25,34,37]. The depth of ion damage increases with ion energy, and it appears that the surface roughening caused using 500 eV ions is not sufficiently repaired during the annealing process to allow the formation of straightened step edges.

Pyramid formation in our data is always coexistent with step edges aligned with the $\left[1\;\bar{1}\;\bar{2}\right]$ and $\left[1\;\bar{1}\;2\right]$ directions; this is evident when using 500 eV Ar^+^ (Figure 6d), and also at low flux with 100 eV Ar^+^ (Figure 2c). In our model, the step edges with (16 × 2) reconstruction were pinned at the perimeter of the pyramids, often as {17 15 1} faceting, in order to form {19 13 1} faceting that contributes to pyramid growth. The (16 × 2) surface reconstruction has a propensity to form relatively long straight rows [23,31]. We suspect that pyramids do not form with 500 eV ions because the step edges do not form in the $\left[1\;\bar{1}\;\bar{2}\right]$ and $\left[1\;\bar{1}\;2\right]$ directions and cannot contribute to the {19 13 1} faceting of the pyramid walls.

Mounds formed with 500 eV ions are likely initiated by Ag clusters, similarly to the pyramids, and they are stable because sputtering at a ridge is minimized. The mounds are smaller than pyramids formed with lower energy ions because they no longer have a contribution of layer-by-layer growth as with the pyramid structures. We suspect that the mounds formed at the apex of pyramids at lower energies are essentially the same as the mounds formed at 500 eV; the minimization of sputtering at the ridge helps with the stability of the pyramids (both during and after nucleation) and makes their height greater than it would be without this effect.

Another effect of ion bombardment that should be addressed is the implantation of Ar^+^ ions and the coincidental creation of Frenkel pairs near the surface. Positive growth through anisotropic adatom migration was found to be the primary method of growth for similar pyramids [5]. Adatoms are likely formed during ion bombardment as a component of Frenkel pairs, and it has been tempting to suggest that the Ehrlich–Schwoebel (ES) barrier may encourage adatom movement towards the top of the {17 15 1} or {19 13 1} faceting sections; this would both provide a contribution to pyramid growth and explain the nucleation of pyramids nearby to other pyramids. We annealed our samples to 800 °C, and another work found a reduction of ES anisotropy on the Ge surface above 250 °C [3]. Due to our high annealing temperature, we suspect that the ES barrier does not induce anisotropy at locations where the step edges are spaced close together, and other studies suggest a similar conclusion [3,19,20,28,29].

### Model for {19 13 1} Faceting

**Figure 7:**
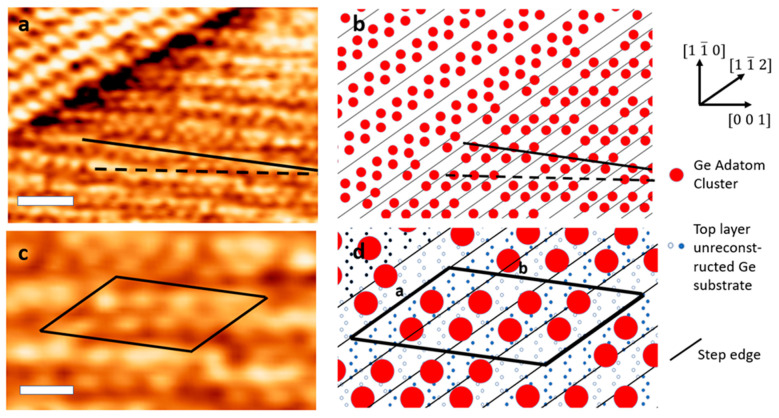
STM images and models of Ge (110) faceting. (**a**) STM topography of {17 15 1} faceting on the left and {19 15 1} faceting on the right. (**b**) Model of {17 15 1} faceting on the left and {19 15 1} faceting on the right. (**c**) STM image of proposed unit cell. (**d**) Model of proposed unit cell. The dotted line in (**a**,**b**) shows the direction of short-range order, while the solid line shows the direction of long-range order. In models (**b**,**d**), the height of the substrate increases by one atomic layer at each step edge moving to the right. Scale bars: (**a**) 5 nm, (**c**) 2 nm. A unit cell of a common superstructure is shown in (**c**,**d**), with lengths a: 34.7 Å and b: 57.7 Å (corresponding to 5√(3/2) a, and √104 a, where a = 5.66 Å).

Figure 7 shows an STM image with {19 13 1} faceting forming a pyramid sidewall, and an accompanying model. In Figure 7a, the upper-left corner consists of {17 15 1} faceting, while the right side shows {19 13 1} faceting on a pyramid wall. Figure 7c is a magnified image of a {19 13 1} section from Figure 7a with a unit cell of a common superstructure outlined. Figure 7b,d show atomic models corresponding to Figure 7a,c respectively. In the models, the step edges shown as horizontal lines increase in height by one atomic step moving to the right. A model for {17 15 1} faceting has already been proposed [23], and the adatom clusters, shown as red dots in the model, are suspected to correspond to a surface reconstruction containing 4–5 Ge atoms on top of the unreconstructed Ge substrate. We observe similar rows of bumps in {19 13 1} faceting. We suspect that each bump observed in STM corresponds to a cluster of Ge adatoms, though we do not resolve the details of this cluster. The closeness of the steps in the {19 13 1} region, and the tight spacing between bumps observed in STM, suggest that these clusters are likely distinct from those in {17 15 1} faceting. Moreover, the top layer of the unreconstructed Ge substrate likely has some perturbation due to relaxation.

The reconstruction on {19 13 1} consists of rows of atomic clusters. The short-range order of clusters runs along the [0 0 1] direction; this is indicated by the dotted lines in Figure 7a,b, and is evident in the magnified image shown in Figure 7c. The long-range order is at a different angle, 8.17° from [0 0 1], as indicated by the thick solid line in Figure 7a,b. An example unit cell of the superstructure containing eight clusters is shown in Figure 7c,d. While this unit cell represents a common observation of structure, there are many irregularities and defects on the surface. For example, in Figure 7a there are some locations where the short-range order is aligned with the long-range order, or where the short-range order runs along more or fewer clusters than shown in the model. There are also locations with missing clusters, in addition to some groups of clusters without order. The model we present represents the majority of the surface, and it also allows for the observed correspondence between the short- and long-range orders.

## 5. Conclusions

Isolated pyramids form during sputter-annealing cycles on the Ge (110) surface. Pyramids have four walls with {19 13 1} faceting and a steep mound at the apex. Pyramids form using Ar^+^ between 200 eV and 400 eV and require Ag to be present on the sample or sample holder. The sputter-annealing cycles cause step edges to be pinned around the base of existing pyramids, while surface layers are locally removed in a layer-by-layer fashion. The inhibition of the migration of step edges at the perimeter of the pyramids is proposed to be the primary component of pyramid growth. As each terrace is removed around the location of the pyramid, the reconstruction at the base of the pyramid converts to {19 13 1} faceting, and the pyramid grows in height by one atomic layer. The absence of pyramids using 500 eV Ar^+^ is suspected to be due to surface damage that is insufficiently removed during the annealing cycles. The surface reconstruction of the {19 13 1} faceting displays bumps associated with atomic clusters similar to those on the {17 15 1} faceting, and a model is provided.

## Figures and Tables

**Figure A1:**
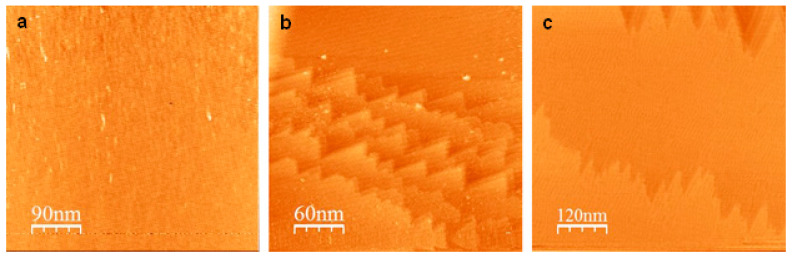
STM topographical images of bare Ge (110) with (**a**) 6 cleaning cycles, (**b**) 14 cleaning cycles, and (**c**) 21 cleaning cycles on a completely new sample holder using 400 eV Ar^+^. Imaging parameters are (**a**) 1 nA, 1 V tip bias; (**b**) 4 nA, 1.5 V tip bias; (**c**) 3 nA, 2 V tip bias.

**Figure A2:**
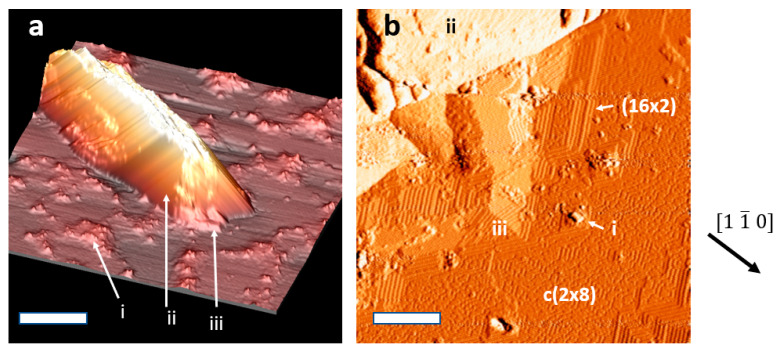
Deposition of ~10 MLs of Ag on Ge (110), annealed to 177 °C, and cooled to RT for imaging. (**a**) A 3D STM topographical image of a 1D Ag island on a Ge (110) substrate. (**b**) Topographical STM image of the boundary of a pyramid showing magnified region “iii” from (**a**). Scale bars: (**a**) 200 nm, (**b**) 40 nm.

## Data Availability

All of the data relevant to this study are provided in the Figures and Appendix A and Appendix B. Further information can be requested from the corresponding author.

## Appendix A. Ar^+^ Cleaning without Ag Present

In order to verify that the presence of Ag is required for pyramid formation, STM measurements and sputter cleaning were performed on a Ge (110) sample with no Ag contamination, using a sample holder that was constructed entirely using new components. All sample preparation and cleaning procedures were the same, except that the sputtering current was measured to be 5 µA at 5 × 10^−6^ torr Ar partial pressure for energy of 200 eV.

For the images shown in Figure A1, the sputtering energy of 400 eV was chosen because it caused the growth of very pronounced pyramids (cf. Figure 4 and Figure 6c) that would easily appear in STM images. Figure A1 shows the results of sputtering after 6, 14, and 21 cycles for (a), (b), and (c), respectively. Different samples were used for the images—(a) was imaged using the first sample, while (b) and (c) were imaged using a second sample. None of these images showed any sign of pyramid formation, confirming that the samples discussed in the main part of the paper needed Ag in order to form nucleation points for the pyramid formation.

## Appendix B. Formation of Large 1D Ag Islands

Following experiments involving the deposition of ~10 MLs of Ag on Ge (110) at room temperature (RT), subsequent annealing to 177 °C, and then cooling to RT, large 1D Ag islands formed on the surface. Figure A2a,b show the Ag island labeled as “ii”. Most of the remaining surface displays bare Ge (110) with c (2 × 8) and (16 × 2) surface reconstructions, as shown in Figure A2b. Interesting exceptions to this observation are as follows:

Figure A2a, “i” shows an example of a cluster of small pyramids. Figure A2b shows a similar isolated small pyramid labeled “i”. At the apex of these pyramids are mounds with a structure that is not well determined. These mounds are often elongated in the $\left[1\;\bar{1}\;0\right]$ direction, which is the same direction as the Ag 1D island growth. We suspect that these mounds contain Ag.

Figure A2a,b location “iii” shows where {19 13 1} faceting is present, which is consistent with the structure of large pyramid walls.

Location “iii”, with {19 13 1} faceting, is the tallest Ge faceting in the local vicinity, and also corresponds with the edge of the 1D Ag island. We suspect that 1D Ag islands preferentially nucleate at locations similar to large pyramid structures. LEEM observations confirmed that 1D Ag islands nucleate at large defects on the surface. Although the defects appeared to be of similar size and shape to the pyramids seen in the STM images, the LEEM images did not provide sufficient resolution to confirm this.
